# Backbone resonance assignment of the third domain of the human staphylococcal nuclease domain-containing protein 1 (SND1)

**DOI:** 10.1007/s12104-026-10267-4

**Published:** 2026-05-16

**Authors:** Shradha Ajith, Liza Cubeddu, Roland Gamsjaeger

**Affiliations:** 1 School of Science, Faculty of Engineering, Computing and Science, Western Sydney University, Penrith, NSW 2751 Australia; 2 School of Life and Environmental Sciences, University of Sydney, Sydney, NSW 2006 Australia

**Keywords:** SND1, Nsp9, SARS-CoV-2, virology

## Abstract

Staphylococcal nuclease and Tudor domain‑containing protein 1 (SND1) is a multifunctional RNA‑binding protein implicated in transcriptional regulation, post‑transcriptional RNA control, oncogenesis, and viral infection. Initially identified as a transcriptional coactivator, SND1 was later established as a component of the RNA‑induced silencing complex, where it contributes to RNA turnover and microRNA regulation. SND1’s diverse activities stem from its modular architecture, comprising four staphylococcal nuclease‑like domains, capable of direct RNA binding, and an extended Tudor domain that together form an integrated RNA‑binding and catalytic platform. This versatility also underlies its role in viral infection: SND1 acts as an m⁶A reader and is exploited by RNA viruses, such as SARS‑CoV‑2. Recent work showed that SND1 depletion, particularly loss of its third structured domain (SN3), reduces recruitment of the viral protein Nsp9 to the 3′ untranslated region of the SARS‑CoV‑2 genome, impairing viral RNA synthesis through a direct SN3–Nsp9 interaction. Here, we report expression, purification, and near‑complete backbone NMR assignments of the SN3 domain of SND1. Secondary structure elements calculated by TALOS-N based on these assignments are in good agreement with the existing crystal structure of SN3. Our data provide an excellent foundation for future structural studies of SND1–RNA complexes and their roles in viral RNA priming in SARS-CoV-2.

## Biological Context

Staphylococcal nuclease and Tudor domain-containing protein 1 (SND1), also known as p100 or Tudor-SN or TSN, is a multifunctional RNA-binding protein that has emerged as a critical regulator of gene expression in cancer, RNA metabolism, and viral infection. SND1 was initially identified as a nuclear transcriptional coactivator, capable of bridging transcription factors to the basal transcription machinery.

Early studies demonstrated that SND1 functions as the coactivator of human EBNA-2 (Epstein-Barr virus nuclear antigen 2) and as a co-activator for STAT6, physically linking STAT6 to RNA polymerase II and enhancing cytokine-responsive transcriptional programs (Callebaut and Mornon 1997; Yang et al. 2002). Later studies have generally established that SND1 plays a central role in post-transcriptional RNA control as it has been identified as a component of the RNA-induced silencing complex (RISC) where SND1 preferentially binds hyper-edited double-stranded RNA and promotes its degradation (Scadden 2005). Biochemical studies have also found that SND1 contributes to microRNA turnover and fine-tuning of RNA stability, thereby shaping post-transcriptional gene regulatory networks (Gutierrez-Beltran et al. 2016; Li et al. 2018). Subsequent work established SND1 as an oncogene, with elevated expression observed across multiple tumour types. SND1 promotes cancer cell proliferation, survival, invasion, and metastasis, in part through transcriptional and post-transcriptional mechanisms (Guo et al. 2014; Ochoa et al. 2018). A major advance came with the identification of the complex it forms with metadherin, MTDH-SND1, which enhances tumour progression by stabilizing oncogenic transcripts and promoting aggressive phenotypes (Guo et al. 2014). Structural characterization of this complex revealed how SND1 contributes to tumour-promoting RNA regulation; pharmacological disruption of its interactions, like the MTDH-SND1 interaction or the SND1-RNA binding, suppresses tumour growth and sensitizes cancer cells to apoptosis-inducing therapies (Lehmusvaara et al. 2022; Shen et al. 2022). Together, these findings placed SND1 at the centre of cancer-associated RNA regulatory networks.

Insights into SND1’s function have been greatly shaped by its distinctive domain architecture. Bioinformatic and structural analyses revealed that SND1 is composed of four staphylococcal nuclease-like (SN) domains followed by an extended Tudor domain within its fifth SN domain (Callebaut and Mornon 1997; Li et al. 2008). High-resolution structural studies demonstrated that these domains cooperate to form an integrated RNA-binding and catalytic platform, with the SN domains contributing to ribonuclease activity and the Tudor domain to protein-protein interaction, such as methylated peptides (Friberg et al. 2009; Li et al. 2008). This modular organization underlies SND1’s ability to engage with a wide range of RNA substrates while still maintaining specificity for particular RNA architectures.

The RNA-binding versatility of SND1 is likewise evident in the context of viral infection. SND1 functions as an m⁶A (N⁶-methyladenosine) RNA reader and is essential for replication of Kaposi’s sarcoma-associated herpesvirus, linking host epi-transcriptomic recognition to viral RNA metabolism (Baquero-Perez et al. 2019). These findings indicate that RNA viruses can exploit host RNA-binding proteins capable of recognizing structured RNA, such as SND1. In a recent study, this concept was reinforced by Schmidt et al. (2023), who showed that depletion of SND1 reduces the presence of the viral non-structural protein Nsp9 at the structurally highly organised 3′ untranslated region of the SARS-CoV-2 genome and consequently diminishes viral RNA synthesis (Cao et al. 2021; Ohyama et al. 2024; Schmidt et al. 2023; Zhao et al. 2020). Importantly, as demonstrated in this recent work (Schmidt et al. 2023), this effect is mediated by the physical interaction between the third domain of SND1 (SN3) and Nsp9.

Although crystal structures of SND1 fragments, including SN3, have been published (Li et al. 2008), no solution structures of any of the first four SN domains - either in their unbound form or in complex with structured RNA - are currently available. In this study, we have expressed and purified SND1 SN3 and have obtained almost complete solution-state backbone resonance assignments of SN3 at 25 °C. These data provide a foundation for determining SND1–RNA structures by NMR, ultimately gaining insights into the molecular mechanism by which SND1 contributes to the priming of viral RNA synthesis in SARS-CoV-2, the essential initial step in viral replication.

## Methods and experiments

### Cloning, protein expression and purification

The third domain of the SND1 protein (SN3; residues 340–496) was commercially obtained and ligated into the BamHI/EcoRI site of expression vector pGEX-6P-1 to generate an N-terminus GST fusion construct. Recombinant expression of GST-tagged SN3 was induced in *E. coli* BL21(DE3) cells by the addition of 1mM IPTG for 20 h at 20 °C. Isotopically labelled ^15^N-SN3 and ^15^N^13^C-SN3 were prepared in a bio-fermenter using the published protocol with 1.25 g/L ^15^NH_4_Cl and 2.5 g/L ^13^C-glucose (Cai et al. 1998). Pelleted cells were lysed by sonication in lysis buffer (10 mM MES pH 6.0, 500 mM NaCl, 3 mM TCEP, 0.1% (v/v) Triton X-100, 0.5 mM PMSF, 1 µg/mL DNase I) and centrifuged at 4 °C. The soluble fraction was bound to Glutathione-Sepharose beads that were washed in lysis buffer, exchanged into wash buffer (10 mM MES pH 6.0, 50 mM NaCl, 3 mM TCEP) and cleaved overnight at 4 °C with the addition of cleavage protein HRV-3 C. Cleaved protein (remaining GPLGS tag at the N-terminus) was eluted using the wash buffer and run through a heparin column with a salt gradient (10 mM MES pH 6.0, 3 mM TCEP, 50 to 1000 mM NaCl). Fractions correlating to the distinct protein peak detected by UV light (280 nm) were collected, concentrated and subjected to size exclusion chromatography (SEC) in a HiLoad™ 16/600 Superdex™ 75 pg column (GE Healthcare) in NMR buffer (25 mM sodium phosphate pH 6.0, 150 mM NaCl, 1 mM DTT. Protein fractions (280 nm) were collected and analysed by SDS-PAGE. The concentration of the protein was determined using the calculated theoretical molar extinction coefficient at 280 nm (8940 M^− 1^ cm^− 1^) for SN3.

### NMR spectroscopy & data processing

Purified isotopically labelled ^15^N-SN3 (390 µM) and ^15^N^13^C-SN3 (382 µM) samples were prepared in NMR buffer in the presence of 10% deuterium oxide (D_2_O) and 20 µM 4,4-dimethyl-4-silapentane-1-sulfonic acid (DSS). Spectra were acquired on a Bruker Avance III 800 MHz spectrometer at 25 °C. The spectra recorded to obtain protein backbone chemical shift assignments include ^15^N-HSQC, CBCA(CO)NH, HNCACB, HNCO, HN(CA)CO, and ^15^N-NOESY. All spectra were processed using TOPSPIN4.5 (Bruker, Biospin) with reference to the chemical shift of DSS (0 ppm) and assignments were obtained using Sparky (T. D. Goddard and D.G. Kneller, University of California at San Francisco). TALOS-N (Shen and Bax 2013) was used to calculate secondary structure elements of SN3 and compared to the existing crystal structure (PDB 3BDL).

### Extent of assignments and data deposition

**Fig. 1:**
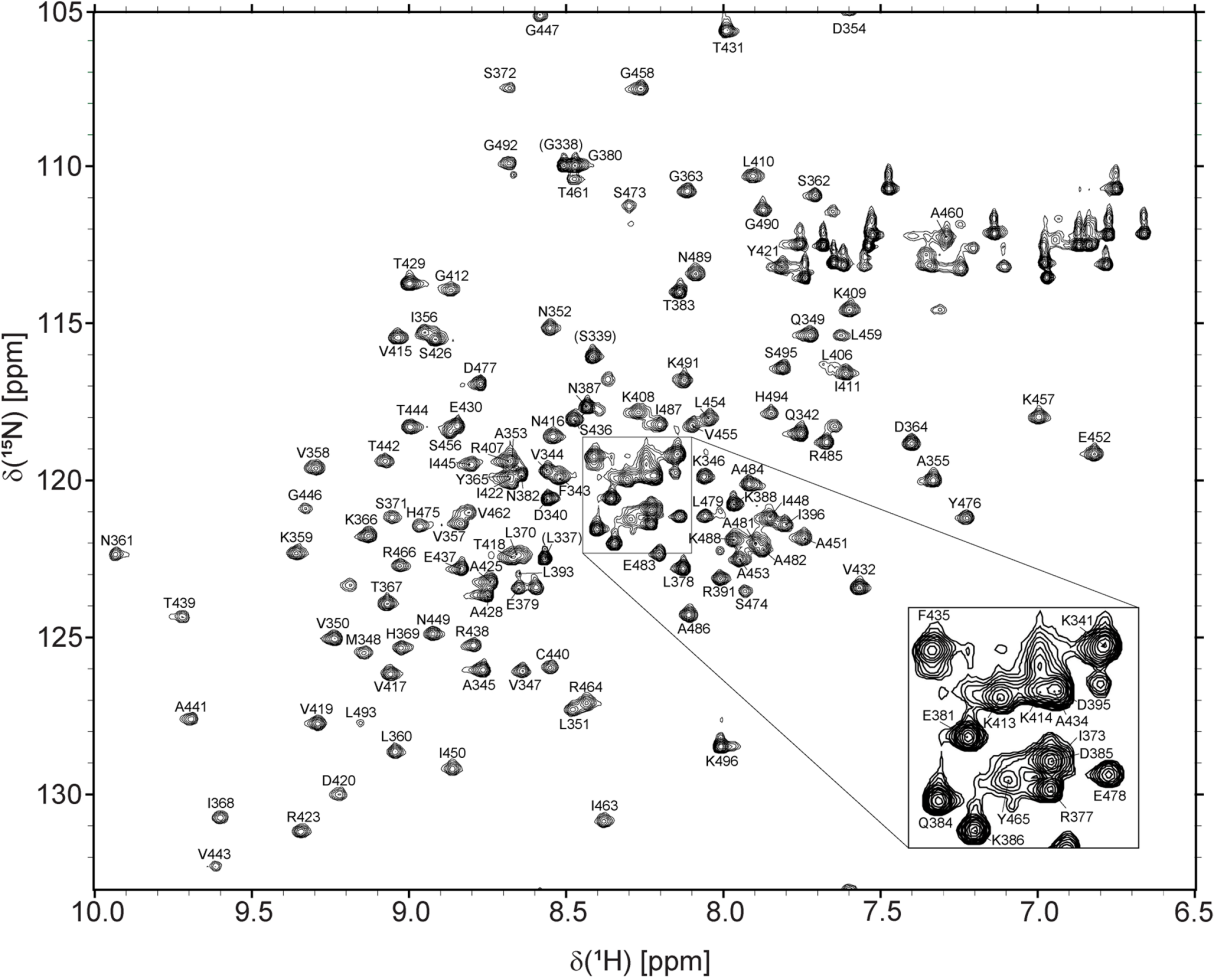
^15^N-HSQC spectrum of SND1 SN3 domain (340–496; ~400 µM) with three additional residues from HRV-3 C cleavage (L337, G338 and S339; all indicated in brackets) showing backbone amide resonances. Note that for clarity, residues 341, 373, 377, 381, 384, 385, 386, 395, 413, 414, 434, 435, 465 and 478 located in the centre of the spectrum are not labelled and are shown as in inset in the bottom right of the Figure. The spectrum was recorded at a proton resonance frequency of 800 MHz at 25 °C in 25 mM sodium phosphate pH 6.0, 150 mM NaCl and 1 mM DTT

The protein construct used in this study (residues 340–496 excluding the expression tag; numbering is based on (Li et al. 2018) comprised the full structured third domain of SND1 (SN3). The ^1^H^15^N HSQC spectrum of folded SN3 is shown in Fig. 1. Note that we are unable to locate and assign residues 398–405 within the α‑helix spanning residues 398–410 as well as the disordered loop encompassing residues 467–472 (sequence QDDDQR) resulting in an overall completeness of 86% (83% for ^15^N, 88% for ^13^Cα, 86% for ^13^Cβ and 86% for ^13^CO).

**Fig. 2:**
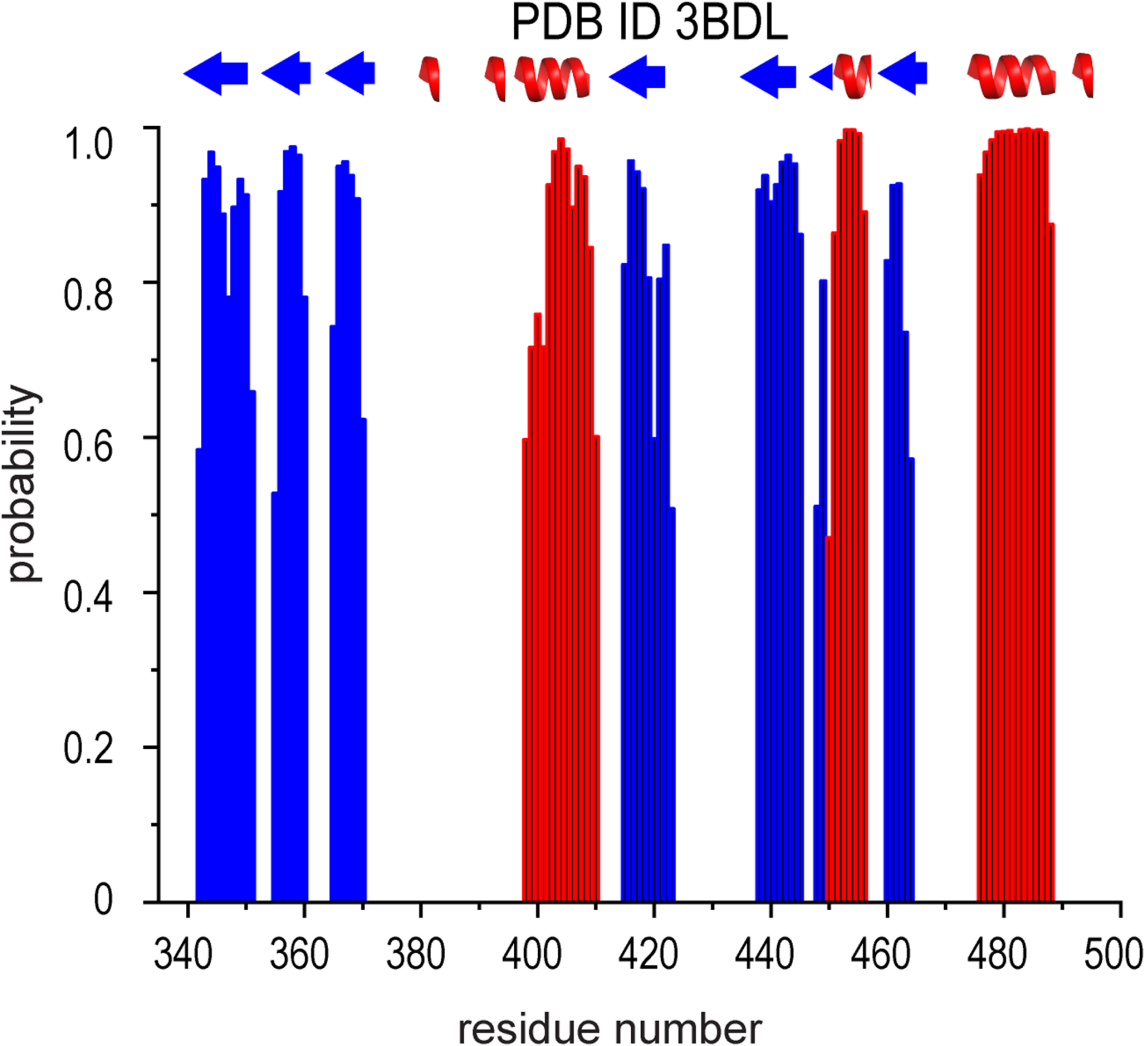
Secondary structure prediction of SN3 based on backbone chemical shifts. TALOS-N prediction of α-helices (red) and ß-sheets (blue) in the SN3 structure based on NMR backbone chemical shift data. The top of the Figure depicts a schematic of the secondary structure elements as observed in the published X-ray structure (PDB 3BDL)

Attempts to perform additional NMR experiments to obtain side‑chain chemical shift assignments were unsuccessful, as the resulting spectra lacked sufficient quality—most likely due to resonance signals undergoing intermediate exchange. All chemical shifts have been verified and deposited into the BMRB (http://www.bmrb.wisc.edu) under the accession number 53,616. In addition, we have carried out TALOS-N calculations (https://spin.niddk.nih.gov/bax/nmrserver/talosn/) (Shen and Bax 2013) (Fig. 2). As seen in the Figure, the predicted secondary structure elements are in excellent agreement with our assigned chemical shifts. We have also attempted to use CS-Rosetta (Lange et al. 2012; Shen et al. 2010; Shen et al. 2008; Shen et al. 2009) to calculate a structure of SN3. However, although the predicted helices and β-sheets in the top 10 CS-Rosetta models agreed well with those in the crystal structure, the models themselves did not converge, indicating that CS-Rosetta was unable to reproduce the structure observed by X-ray crystallography (data not shown).

In summary, our data provide an excellent foundation for future structural studies of SND1–RNA complexes and their roles in viral RNA priming in SARS‑CoV‑2, offering the framework needed to dissect the molecular determinants of RNA recognition and potentially guide further targeted investigations into how SND1 contributes to viral replication.

## Data Availability

Assignments for the SND1 SN3 domain have been deposited in the BMRB under accession number 53616.
